# S-acylation of Sprouty and SPRED proteins by the S-acyltransferase zDHHC17 involves a novel mode of enzyme–substrate interaction

**DOI:** 10.1016/j.jbc.2022.102754

**Published:** 2022-11-25

**Authors:** Liam Butler, Carolina Locatelli, Despoina Allagioti, Irina Lousa, Kimon Lemonidis, Nicholas C.O. Tomkinson, Christine Salaun, Luke H. Chamberlain

**Affiliations:** 1 Strathclyde Institute of Pharmacy and Biomedical Sciences, University of Strathclyde, Glasgow, United Kingdom; 2 UCIBIO, REQUIMTE, Laboratory of Biochemistry, Faculty of Pharmacy, University of Porto, Porto, Portugal; 3 School of Molecular Biosciences, College of Medical Veterinary and Life Sciences, University of Glasgow, Glasgow, United Kingdom; 4 Department of Pure and Applied Chemistry, University of Strathclyde, Glasgow, United Kingdom

**Keywords:** acyltransferase, protein acylation, protein palmitoylation, protein–protein interaction, zDHHC enzymes, zDHHC17, ankyrin repeat domain, Sprouty-2, SPRED

## Abstract

S-acylation is an essential post-translational modification, which is mediated by a family of 23 zDHHC enzymes in humans. Several thousand proteins are modified by S-acylation; however, we lack a detailed understanding of how enzyme–substrate recognition and specificity is achieved. Previous work showed that the ankyrin repeat domain of zDHHC17 (ANK17) recognizes a short linear motif, known as the zDHHC ANK binding motif (zDABM) in substrate protein SNAP25, as a mechanism of substrate recruitment prior to S-acylation. Here, we investigated the S-acylation of the Sprouty and SPRED family of proteins by zDHHC17. Interestingly, although Sprouty-2 (Spry2) contains a zDABM that interacts with ANK17, this mode of binding is dispensable for S-acylation, and indeed removal of the zDABM does not completely ablate binding to zDHHC17. Furthermore, the related SPRED3 protein interacts with and is efficiently S-acylated by zDHHC17, despite lacking a zDABM. We undertook mutational analysis of SPRED3 to better understand the basis of its zDABM-independent interaction with zDHHC17. This analysis found that the cysteine-rich SPR domain of SPRED3, which is the defining feature of all Sprouty and SPRED proteins, interacts with zDHHC17. Surprisingly, the interaction with SPRED3 was independent of ANK17. Our mutational analysis of Spry2 was consistent with the SPR domain of this protein containing a zDHHC17-binding site, and Spry2 also showed detectable binding to a zDHHC17 mutant lacking the ANK domain. Thus, zDHHC17 can recognize its substrates through zDABM-dependent and/or zDABM–independent mechanisms, and some substrates display more than one mode of binding to this enzyme.

S-Acylation is a widespread post-translational modification of cellular proteins involving the reversible attachment of fatty acyl chains onto cysteine residues (1, 2). In humans, this process is performed by 23 distinct “zDHHC” enzyme isoforms (3, 4, 5, 6). All zDHHC enzymes are polytopic membrane proteins and share a conserved catalytic domain (the zinc finger DHHC cysteine-rich domain) positioned at the cytosol–membrane interface, which mediates the S-acylation of cysteine residues in close membrane proximity (in both soluble and transmembrane proteins) (7, 8). Current knowledge about enzyme–substrate specificity in S-acylation pathways is very limited, and it is important to delineate how zDHHC enzymes recognize their substrates and to reveal the identity of the substrate networks of individual zDHHC enzymes (9).

It is likely that some zDHHC enzymes mediate substrate S-acylation without requiring selective interactions with their substrates (10). Here, the intrinsic high activity of certain zDHHC enzymes may allow the transfer of acyl chains from the autoacylated enzyme intermediate to nearby cysteines in other proteins without a direct enzyme–substrate interaction. Specificity in S-acylation reactions mediated by these high-activity/low-specificity enzymes is likely determined by the substrate and accessibility of its cysteines, that is, only proteins with appropriately positioned and reactive cysteines can be modified by this low-specificity S-acylation process. This process might be important to allow the modification of a large and diverse array of transmembrane proteins by cellular S-acylation enzymes. However, in addition to these low-specificity enzyme–substrate interactions, other zDHHC enzymes have been reported to recognize specific features of proteins to facilitate the S-acylation of a selective group or network of substrate proteins (9). These high-specificity enzyme–substrate interactions may be important, for example, in recruiting soluble proteins to membranes, facilitating their subsequent S-acylation. Two zDHHC enzymes that appear to operate as high-specificity enzymes are zDHHC17 and zDHHC13. These related enzyme isoforms are the only mammalian zDHHC enzymes that contain an N-terminal ankyrin repeat (ANK) domain that has been shown to mediate recognition and binding of a number of substrates. The importance of this ANK domain for substrate recognition was first reported for huntingtin (HTT) (11) and later work demonstrated that the ANK domains of zDHHC17 (ANK17) and zDHHC13 (ANK13) also mediate interaction with other substrate proteins, such as SNAP25 (synaptosomal-associated protein of 25 kDa) and CSP (cysteine-string protein) (12). Interestingly, in contrast to zDHHC17, the interaction of zDHHC13 with SNAP25 and CSP does not result in their S-acylation perhaps due to conformational constraints that limit access of the catalytic domain of zDHHC13 to specific cysteine residues in these proteins when they are bound to ANK13 or alternatively the unique DQHC motif of zDHHC13 may limit the substrate specificity of this enzyme (12, 13). Further analyses of the interaction of substrates including SNAP25, CSP, and HTT with zDHHC17 identified a consensus [VIAP][VIT]*XX*QP motif that interacts with ANK17 (14). This consensus sequence is referred to as the “zDHHC ankyrin-repeat binding motif” (zDABM) and it is required to be present in a cytosolic unstructured region of the protein to allow binding to ANK17 (14).

A high-resolution crystal structure of ANK17 in complex with a peptide fragment containing the zDABM of SNAP25 (amino acids 111-GVVASQPARV-120; zDABM is underlined) was recently solved, highlighting the specific residues in ANK17 that interact with zDABM sequences (15). Specifically, Asn-100 (N100) and Trp-130 (W130) were shown to be essential for zDABM binding. W130, through its aromatic ring, establishes key contacts with the highly conserved proline residue (P117) of the SNAP25 zDABM, whereas N100 forms hydrogen bonds with Val-113 (V113) (15). In addition, Tyr-67 (Y67) associates *via* van der Waals interactions with Val-112 (V112) in the SNAP25 peptide, whereas Glu-89 and Asp-122 (E89/D122) in ANK17 establish hydrogen bonds with Gln-116 (Q116) in SNAP25 (15).

By identifying proteins that contain a cytosolic and unstructured zDABM and subsequently validating interactions with ANK17 in peptide arrays, we previously identified a large number of potential novel interactors of zDHHC17, which included members of the Sprouty (Spry) and SPRED protein families (16). Interestingly, the interaction of Spry and SPRED proteins with zDHHC17 was previously reported in a yeast two-hybrid screen (17) and Spry2 and zDHHC17 were shown to interact in a BioPlex interactome study (18).

We recently showed that S-acylation by zDHHC enzymes including zDHHC17 plays a key role in regulating both the stability and localization of Spry2 (19). This study showed that Spry2 turnover was reduced when the protein is S-acylated and further showed that the protein failed to accumulate at the plasma membrane when S-acylation is blocked. Sprouty and SPRED proteins contain a conserved cysteine-rich Sprouty (SPR) domain, which in Spry2 contains 26 cysteines. S-acylation of Spry2 by zDHHC17 depends on two key cysteines in the SPR domain: Cys-265 and Cys-268. The finding that S-acylation regulates Spry2 stability and localization is important because these are two fundamental properties related to the relative activities of cellular proteins, thus emphasizing the importance of Spry2 S-acylation. Indeed, as Spry2 (and other Sprouty and SPRED proteins) is a potential tumor suppressor protein that is downregulated in many different tumor types (20), targeting S-acylation may present a viable approach to enhance Spry2 levels and restore optimal growth factor signaling.

The aim of this study was to investigate the interaction of Spry2 with zDHHC17 to determine if recognition and subsequent S-acylation occurred by the same pathway/mechanism described for SNAP25. This interaction was of particular interest as the BioPlex interactome study found that the endogenous proteins exist in a complex in cells. Our results identify a novel mode of substrate binding and S-acylation by zDHHC17 for Spry2 and SPRED3 and add new insight into mechanisms of substrate recognition by zDHHC enzymes.

## Results

### Disruption of the zDABM-binding site in the ANK domain of zDHHC17 does not affect S-acylation of Spry2

Our recent study showed that S-acylation of the cysteine-rich domain of Spry2 is important for both the intracellular localization and stability of this protein and that zDHHC17 is the main candidate enzyme to mediate Spry2 S-acylation (19). Furthermore, we showed that a zDABM peptide from Spry2 (containing Pro-154 at position 6 of the IIRVQP zDABM) can interact with ANK17 (16). Indeed, an unbiased wide-scale cellular interactome study also reported an interaction between endogenously expressed Spry2 and zDHHC17 (18).

Verardi *et al.* (2017) (15) previously reported that Asn-100 (N100) and Trp-130 (W130) are critical for the interaction of ANK17 with zDABM sequences, and alanine substitutions at these positions block SNAP25 S-acylation by zDHHC17. In addition, alanine substitution of Pro-117 of the SNAP25 zDABM severely impairs both binding to and S-acylation by zDHHC17 (14, 21).

**Figure 1:**
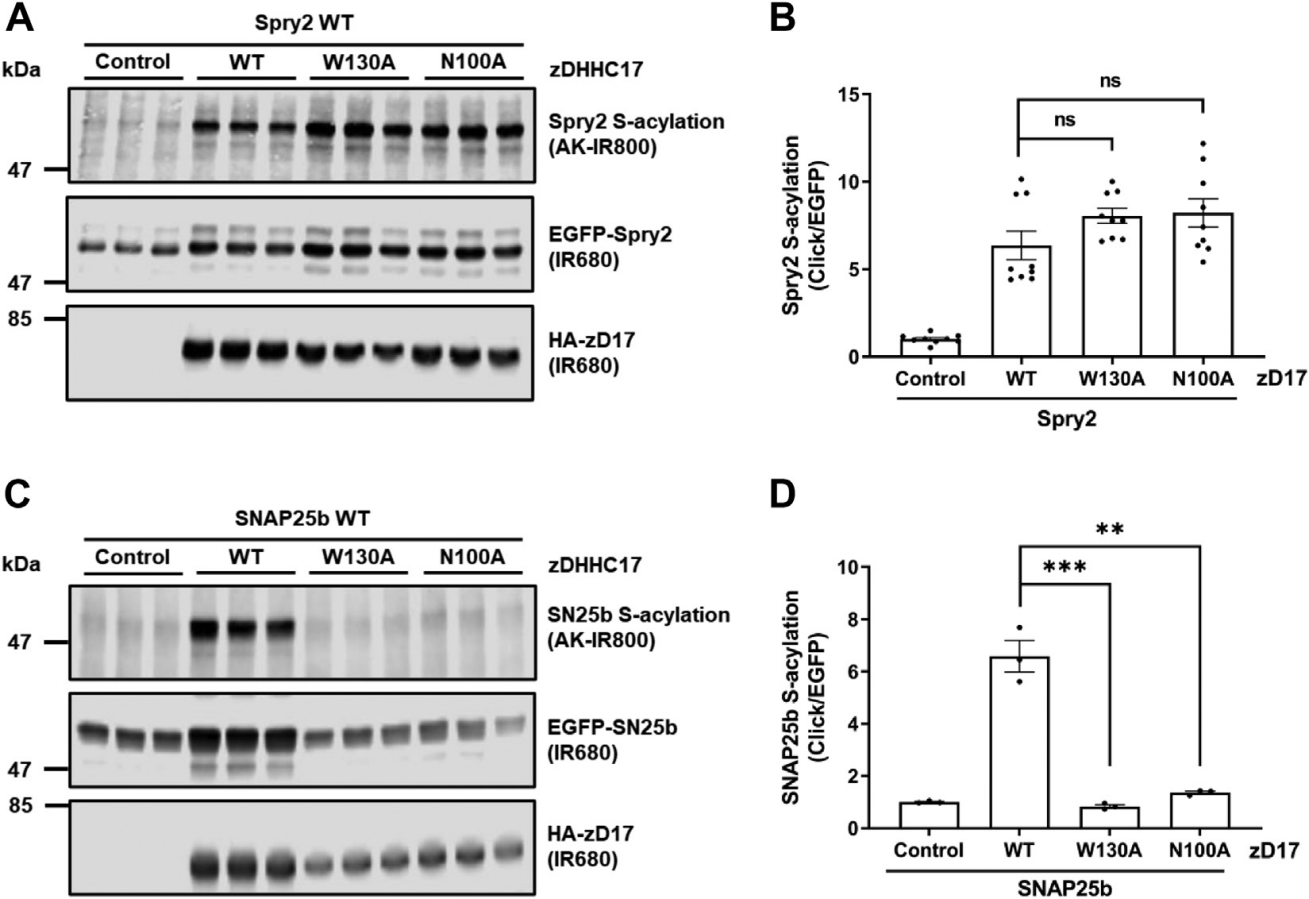
**S-acylation of Spry2 by zDHHC17 does not require Trp-130 or Asn-100.** HEK293T cells were transfected with plasmids encoding either EGFP-tagged Spry2 or SNAP25b, together with either pEF-BOS-HA (referred to as “control” in the figure), HA-tagged zDHHC17 WT, zDHHC17 W130A, or zDHHC17 N100A. Cells were incubated with 100 μM palmitic acid azide for 4 h and labeled proteins reacted with alkyne (AK) IRdye-800 nm. EGFP- and HA-tagged proteins were labeled by immunoblotting using IRdye-680 nm secondary antibodies. *A*, representative images showing Spry2 S-acylation (*top; AK-IR800*) and Spry2 levels (*middle; IR680*) detected on the same immunoblot. For zDHHC17, HA (*bottom; IR680*) was revealed for the same samples on a different immunoblot. The positions of the molecular weight markers are shown on the *left* of all blots. *B*, graph showing mean Spry2 S-acylation levels after normalization. Error bars represent ± SEM; each replicate is shown with *filled circles* (n = 9 different cell samples for each condition). Unpaired *t* test was used to detect significant differences compared to the WT zDHHC17 samples; ns denotes nonsignificance (*p* > 0.05). *C*, representative image showing SNAP25b S-acylation (*top; AK-IR800*), SNAP25b levels (*middle; IR680*), and zDHHC17 levels (*bottom; IR680*). The positions of the molecular weight markers are shown on the *left* of all immunoblots. *D*, graph showing mean SNAP25b S-acylation levels after normalization. Error bars represent ± SEM; each replicate is shown with *filled circles* (n = 3 different cell samples for each condition). Unpaired *t* test was used to detect significant differences compared to the WT zDHHC17 samples (∗∗∗ denotes *p* < 0.001 and ∗∗ denotes *p* < 0.01).

To determine if S-acylation of Spry2 occurs *via* a similar mechanism as described for SNAP25, we investigated the importance of N100 and W130 of ANK17 for Spry2 S-acylation by zDHHC17. Click chemistry S-acylation assays unexpectedly showed that Spry2 was S-acylated by the zDHHC17 N100A and W130A mutants to a level similar to the WT enzyme (Fig. 1*A*). In contrast, neither zDHHC17 N100A nor W130A mutant was able to efficiently S-acylate SNAP25 (Fig. 1*C*), consistent with previous work (15). These data suggest that the canonical substrate-binding pocket in the ANK17 is only required for the S-acylation of a subset of zDHHC17 substrates.

### S-acylation of Spry2 by zDHHC17 does not require a zDABM

**Figure 2:**
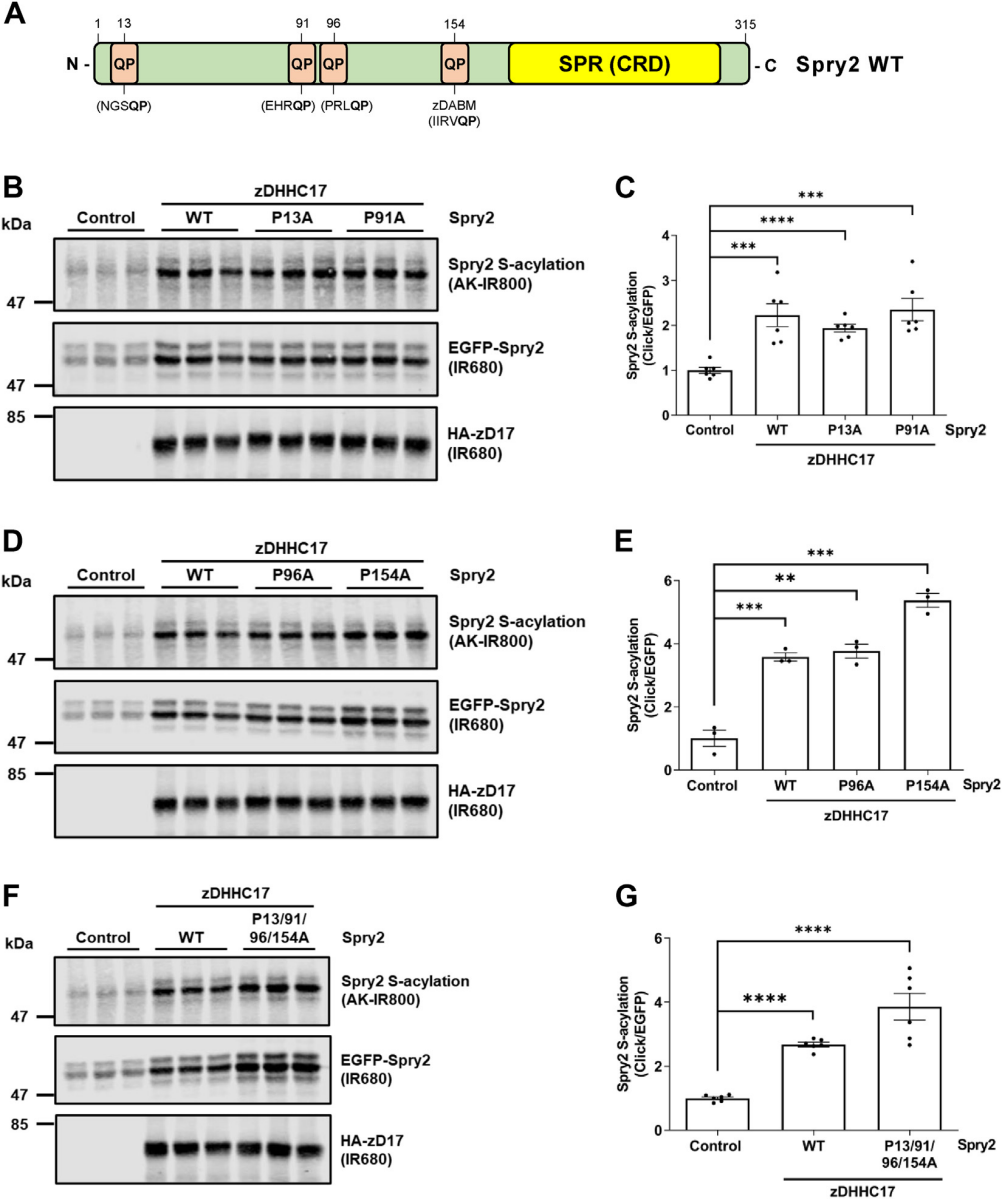
**S-acylation of Spry2 by zDHHC17 does not require zDABM sequences.***A*, schematic representation of mouse Spry2 protein. All QP dipeptides, which were mutated into “QA,” are shown; the zDABM (IIRVQP) containing proline-154 is indicated. All constructs have EGFP tags appended at the N terminus. SPR denotes the Sprouty domain, which is also referred to as CRD for Cysteine-rich domain. *B*–*G*, HEK293T cells were transfected with a plasmid encoding EGFP-tagged Spry2 WT, together with either pEF-BOS-HA (referred to as “control” in the figure) or HA-tagged zDHHC17. Plasmids encoding EGFP-tagged Spry2 P13A, Spry2 P91A, Spry2 P96A, Spry2 P154A, and Spry2 P13/91/96/154A mutants were cotransfected with HA-tagged zDHHC17. Cells were incubated with 100 μM palmitic acid azide for 4 h and labeled proteins reacted with alkyne (AK) IRdye-800 nm. EGFP- and HA-tagged proteins were labeled by immunoblotting using IRdye-680 secondary antibodies. *B*, *D* and *F*, representative images showing Spry2 S-acylation (*top; AK-IR800*) and Spry2 levels (*middle; IR680*) detected on the same immunoblot. For zDHHC17, HA (*bottom; IR680*) was revealed for the same samples on a different immunoblot. The position of the molecular weight markers are shown on the *left* side of all immunoblots. *C*, *E* and *G*, graphs showing mean Spry2 S-acylation levels after normalization. Error bars represent ± SEM; each replicate is shown with *filled circles* (n = 3 or 6, different cell samples for each condition). For clarity, only relevant statistical analysis is shown in the figure. Unpaired *t* test was used to detect significant differences compared to the control sample (∗∗∗∗ denotes *p* < 0.0001, ∗∗∗*p* < 0.001, ∗∗*p* < 0.01). Not shown in the figure: S-acylation of Spry2 P13A, P91A, or P96A *versus* Spry2 WT was not significant (*p* > 0.05); S-acylation of both Spry2 mutants containing the P154A substitution was significantly different from Spry2 WT (P154A was *p* < 0.01 and P13/91/96/154A was *p* < 0.05).

The results in Figure 1 suggest that the interaction of zDABM sequence(s) of Spry2 with the ANK domain of zDHHC17 may not be required for S-acylation. To confirm this directly, we tested if disruption of the zDABM of Spry2 affects its S-acylation by zDHHC17. In peptide-array experiments, the identified zDABM of Spry2, which binds to ANK17 was shown to include Pro-154 (P154) (16). As the proline residue in zDABM sequences is critical for interaction with ANK17, we generated a Spry2 P154A mutant. We also substituted an additional three proline residues that are present in QP sequences (Pro-13, Pro-91, and Pro-96) (Fig. 2*A*), even though none of these QP dipeptides are present in sequences that conform to the canonical zDABM consensus. In addition, a Spry2 quadruple proline mutant (P13A/P91A/P96A/P154A) was also generated.

These EGFP-tagged Spry2 proline mutants were coexpressed in HEK293T cells with HA-tagged zDHHC17, whilst EGFP-tagged Spry2 WT was expressed together with either pEF-BOS-HA (negative control) or HA-tagged zDHHC17. S-acylation was examined by click chemistry, and Figure 2 (panels B-G) shows that all proline mutants were efficiently S-acylated by zDHHC17, confirming that S-acylation is independent of zDABM sequences in Spry2. Indeed, upon zDHHC17 coexpression in HEK293T cells, both P154A and P13A/P91A/P96A/P154A mutants displayed slightly increased S-acylation levels, compared to WT Spry2. Overall, the finding that zDABM sequences are dispensable for Spry2 S-acylation is consistent with the results obtained with the zDHHC17 W130A and N100A mutants (Fig. 1) and suggest that Spry2 S-acylation may involve an alternative, zDABM-independent interaction with zDHHC17.

### The zDABM of Spry2 is dispensable for S-acylation and intracellular targeting of EGFP-Spry2 in PC12 cells

**Figure 3:**
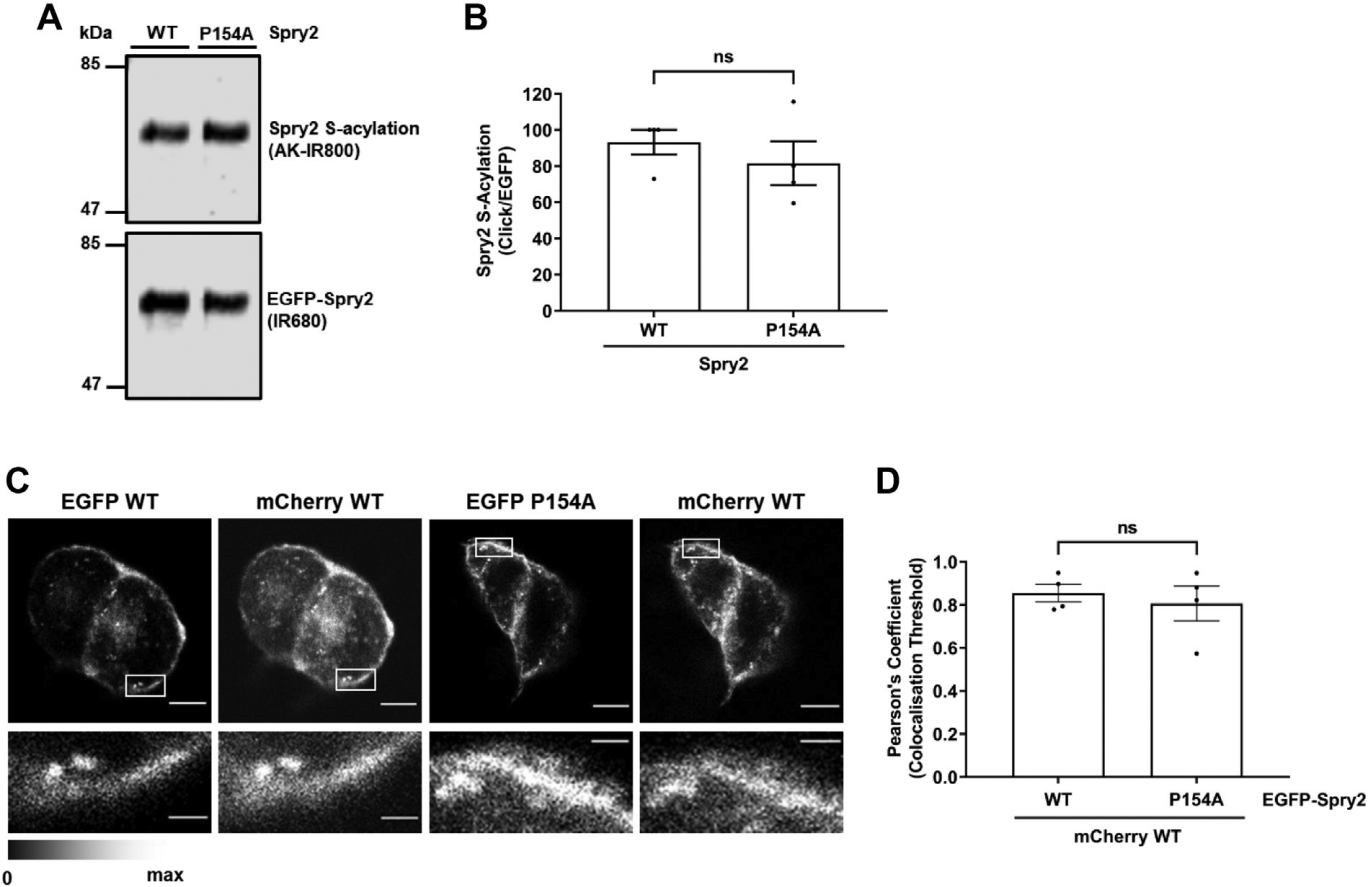
**Mutation of P154 in Spry2 does not affect S-acylation or localization in PC12 cells.***A*, PC12 cells were transfected with either EGFP-Spry2 WT or Spry2 P154A. Cells were incubated with 100 μM palmitic acid azide for 4 h. After cell lysis, labeled proteins were incubated with agarose beads conjugated to an EGFP antibody and later reacted with alkyne (AK) IRdye-800 nm. Representative images showing Spry2 S-acylation (*top panel; IR800*) and Spry2 expression levels (*bottom panel; IR680*) detected on the same immunoblot. The position of the molecular weight markers are shown on the *left* side of the immunoblots. *B*, graph showing mean Spry2 S-acylation levels after normalization. Error bars represent ± SEM; *filled circles* represent independent experiments (n = 4, from three independent experiments). An unpaired *t* test was used to compare S-acylation of Spry2 WT and the P154A mutant (ns denotes nonsignificance *i.e. p* > 0.05). *C*, confocal imaging of PC12 cells cotransfected with plasmids encoding EGFP-Spry2 WT or P154A mutant, together with mCherry-Spry2 WT. Representative images for mCherry and EGFP proteins are shown in the figure (*upper panels*) as well as magnified images of the indicated areas for both channels (*bottom panels*). The scale bars represent 5 μm for upper full images and 1 μm for the lower magnified images. *D*, graph showing Pearson’s correlation coefficient (Rtot). Each bar shows mean values of Rtot ± SEM; *filled circles* represent individual images. Results were analyzed by unpaired *t* test (ns denotes nonsignificance *i.e. p* > 0.05, n = 4).

Our previous work has shown that zDHHC17 substrates are effectively S-acylated in PC12 cells without coexpression of recombinant zDHHC17 (21, 22). We thus investigated whether the P154A mutant of Spry2 can be endogenously S-acylated in neuroendocrine PC12 cells and whether it displays a similar localization as WT Spry2. Click chemistry analysis of the S-acylation status of immunopurified Spry2 P154A showed that this mutant is S-acylated to a similar level as WT Spry2 in PC12 cells (Fig. 3*A*).

Since Spry2 P154A was efficiently S-acylated in PC12 cells, we next examined its subcellular distribution by confocal microscopy. To do this, PC12 cells were cotransfected with EGFP-Spry2 WT or EGFP-Spry2 P154A mutant, together with mCherry-Spry2 WT. In all cases, a fraction of Spry2 proteins was consistently observed at the plasma membrane of cells (Fig. 3*C*). Furthermore, EGFP-Spry2 P154A colocalized with coexpressed mCherry-Spry2 WT both visually and quantitatively (Fig. 3, *C* and *D*). Thus, the zDABM sequence of Spry2 is dispensable for both S-acylation and plasma membrane targeting in PC12 cells. This is in direct contrast to SNAP25, which displays a loss of plasma membrane targeting in PC12 cells when the corresponding zDABM sequence is disrupted (23). Altogether, these analyses further confirm that zDABM sequences are dispensable for Spry2 S-acylation.

### Spry2 truncation mutants containing the SPR domain but lacking the zDABM display reduced binding to zDHHC17 but effective S-acylation

**Figure 4:**
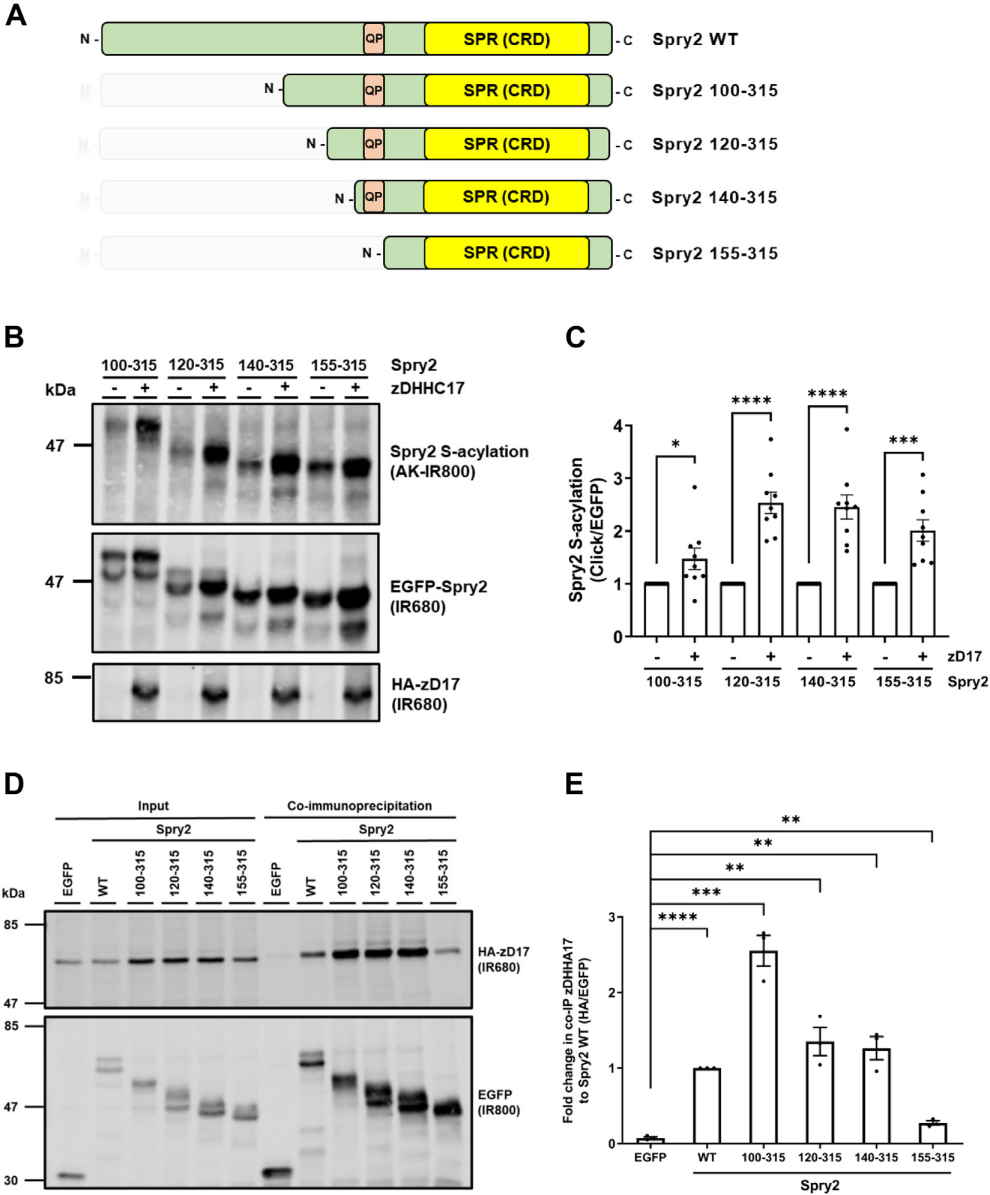
**Residues 155–315 of Spry2, which include the SPR domain, are sufficient for binding to, and S-acylation by, zDHHC17.***A*, schematic of the Spry2 constructs employed in click chemistry and co-immunoprecipitation assays: Spry2 100 to 315, 120 to 315, 140 to 315, and 155 to 315 of the mouse sequence (UniprotKB-Q9QXV8). All constructs have EGFP tags appended at the N terminus. Position of the zDABM containing proline-154 is denoted by “QP;” SPR denotes the Sprouty domain, which is also referred to as CRD (Cysteine-rich domain). *B*, HEK293T cells were transfected with plasmids encoding EGFP-tagged Spry2 100 to 315, Spry2 120 to 315, Spry2 140 to 315, or Spry2 155 to 315, together with either pEF-BOS-HA (referred to as “-” in the figure) or HA-zDHHC17 (referred to as “+” in the figure). Cells were incubated with 100 μM palmitic acid azide (C16:0-azide) for 4 h and labeled proteins reacted with alkyne (AK) IRdye-800 nm. EGFP- and HA-tagged proteins were labeled by immunoblotting using IRdye-680 secondary antibodies. Representative images showing Spry2 S-acylation (*top; AK-IR800*), Spry2 levels (*middle; IR680*), and zDHHC17 levels (*bottom; IR680*), detected on the same immunoblot. The positions of the molecular weight markers are shown on the *left* side of all immunoblots. *C*, graph showing mean Spry2 S-acylation levels after normalization against the corresponding control samples (pEF-BOS-HA). Error bars represent ± SEM; each replicate is shown with *filled circles*. Differences were analyzed by unpaired *t* test (∗∗∗∗ denotes *p* < 0.0001, ∗∗∗*p* < 0.001, ∗*p* < 0.05) (n = 9, for three independent experiments). *D*, HEK293T cells were cotransfected with HA-tagged zDHHA17 (a catalytically inert form of the enzyme) along with plasmids encoding for EGFP-tagged Spry2 100 to 315, Spry2 120 to 315, Spry2 140 to 315, and Spry2 155 to 315, or EGFP alone (as a control). Cell lysates were incubated with agarose beads conjugated to an EGFP antibody and coimmunoprecipitated proteins were analyzed by immunoblotting. Representative images showing zDHHA17 (*top; IR680*) and Spry2 (*bottom; IR800*) levels in the input and immunoprecipitated samples detected on the same immunoblot. The positions of the molecular weight markers are shown on the *left* side of all immunoblots. *E*, graph showing the mean fold change in coimmunoprecipitated zDHHA17 levels after normalization against Spry2 WT. Error bars represent ± SEM; each replicate is shown with *filled circles*. Differences were analyzed by unpaired *t* test compared to the EGFP control (∗∗∗∗ denotes *p* < 0.0001, ∗∗∗*p* < 0.001, ∗∗*p* < 0.01, n = 3 from three independent experiments).

As S-acylation of Spry2 by zDHHC17 occurs through a mechanism independent of zDABM interaction with the previously reported binding site in ANK17 (involving N100 and W130), we further explored the regions of Spry2 that are required for S-acylation. To do this, a series of N-terminal truncation mutants were synthesized (100–315, 120–315, 140–315 and 155–315) (see Fig. 4*A*) and coexpressed with zDHHC17 in HEK293T cells. Click chemistry analysis of S-acylation showed that all four truncation mutants were S-acylated by zDHHC17 (Fig. 4*B*), including the 155 to 315 mutant that lacks the zDABM. We further investigated the interaction of these constructs with zDHHC17 by immunoprecipitating the EGFP-tagged Spry2 proteins and quantifying the amount of coprecipitated zDHHC17. For this experiment, we used a catalytically dead mutant of zDHHC17 to prevent S-acylation–mediated changes in Spry2 expression (19). Figure 4*D* shows that the 100 to 315, 120 to 315, and 140 to 315 mutants all coprecipitated zDHHC17 to a similar or greater level than full-length Spry2. Interestingly, the 155 to 315 Spry2 mutant captured zDHHC17 to a higher level than the negative control (EGFP) but substantially less than was captured by the other Spry2 proteins. This suggests that even though the zDABM is dispensable for S-acylation, it is nevertheless the major binding site for zDHHC17 in Spry2. As the level of binding of zDHHC17 to the 155 to 315 mutant was higher than the negative control (EGFP), Spry2 is likely to contain a second, lower affinity, zDHHC17-binding site downstream of P154. Incidentally, the 100 to 315 Spry2 mutant appeared to show elevated binding (Fig. 4, *D* and *E*) and lower S-acylation (Fig. 4, *B* and *C*) compared to the 120 to 315 and 140 to 315 mutants. We are currently uncertain if these differences are meaningful or a consequence of slightly different folding of these truncation mutants.

### SPRED3 interaction with zDHHC17 involves the cysteine-rich SPR domain

**Figure 5:**
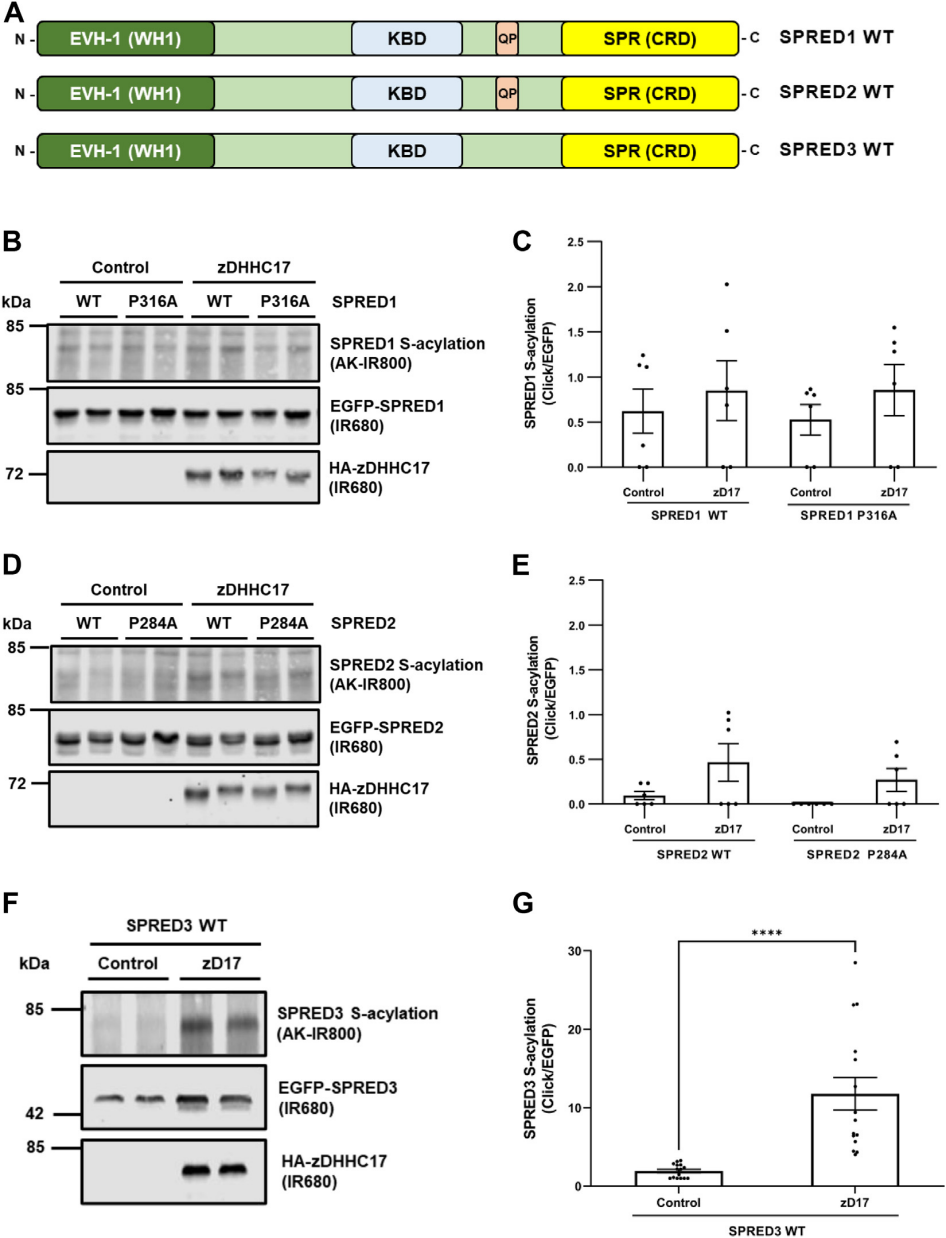
**zDHHC17 is active against SPRED3, which lacks a zDABM.***A*, schematic diagram highlighting the domain structure of mammalian SPRED1/2/3. EVH-1, Ena/VASP (Enabled/vasodilator-stimulated phosphoprotein) Homology 1 domain, also known as WH1; WASP, Wiskott–Alsrich Syndrome Protein homology 1 domain; KBD, c-Kit kinase-binding domain; QP, indicates the zDABM position; SPR, Sprouty domain, also referred to as CRD (Cysteine-rich domain). All constructs used also have EGFP tags appended at the N terminus. *B*–*G*, HEK293T cells were transfected with plasmids encoding for either EGFP-tagged SPRED1 WT or P136A mutant; SPRED2 WT or P284A mutant; or SPRED3 WT, together with pEF-BOS-HA (referred to as “control” in the figure) or HA-tagged zDHHC17 WT. Cells were incubated with 100 μM palmitic acid azide for 4 h and labeled proteins reacted with alkyne (AK) IRdye-800 nm. *B*, representative images showing SPRED1 WT and SPRED1 P316A S-acylation (*top; AK-IR800*), SPRED1 levels (*middle: IR680*), and zDHHC17 levels (*bottom; IR680*). *D*, representative images showing SPRED2 WT and SPRED2 P284A S-acylation (*top; AK-IR800*), SPRED2 levels (*middle: IR680*), and zDHHC17 levels (*bottom; IR680*). *F*, representative images showing SPRED3 WT S-acylation (*top; AK-IR800*), SPRED3 levels (*middle: IR680*), and zDHHC17 levels (*bottom; IR680*). *C*, graph showing mean SPRED1 S-acylation levels after normalization. Error bars represent ± SEM; each replicate is shown with *filled circles* (n = 6 from three independent experiments). Differences were analyzed by unpaired *t* test to the control. No significant differences were present. *E*, graph showing mean SPRED2 S-acylation levels after normalization. Error bars represent ± SEM; each replicate is shown with *filled circles* (n = 6 from three independent experiments). Differences were analyzed by unpaired *t* test to the control. No significant differences were present. *G*, graph showing mean SPRED3 S-acylation levels after normalization. Error bars represent ± SEM; each replicate is shown with *filled circles* (n = 15 from six independent experiments). Differences were analyzed by unpaired *t* test compared to the control (∗∗∗∗ denotes *p* < 0.0001).

Alignment of the Sprouty and SPRED proteins shows that SPRED3 is the only isoform that lacks a zDABM (see schematic of SPRED1-3 in Figure 5*A*). We therefore examined if zDHHC17 could S-acylate SPRED3 and compared this with SPRED1/SPRED2, which do contain zDABM sequences. Interestingly, SPRED1 and SPRED2 were found to be poor substrates of zDHHC17 in click chemistry S-acylation assays, and S-acylation of these proteins was not increased by mutation of their zDABMs (Fig. 5, *B–E*). In contrast, SPRED3 was robustly S-acylated by zDHHC17 (Fig. 5, *F* and *G*).

**Figure 6:**
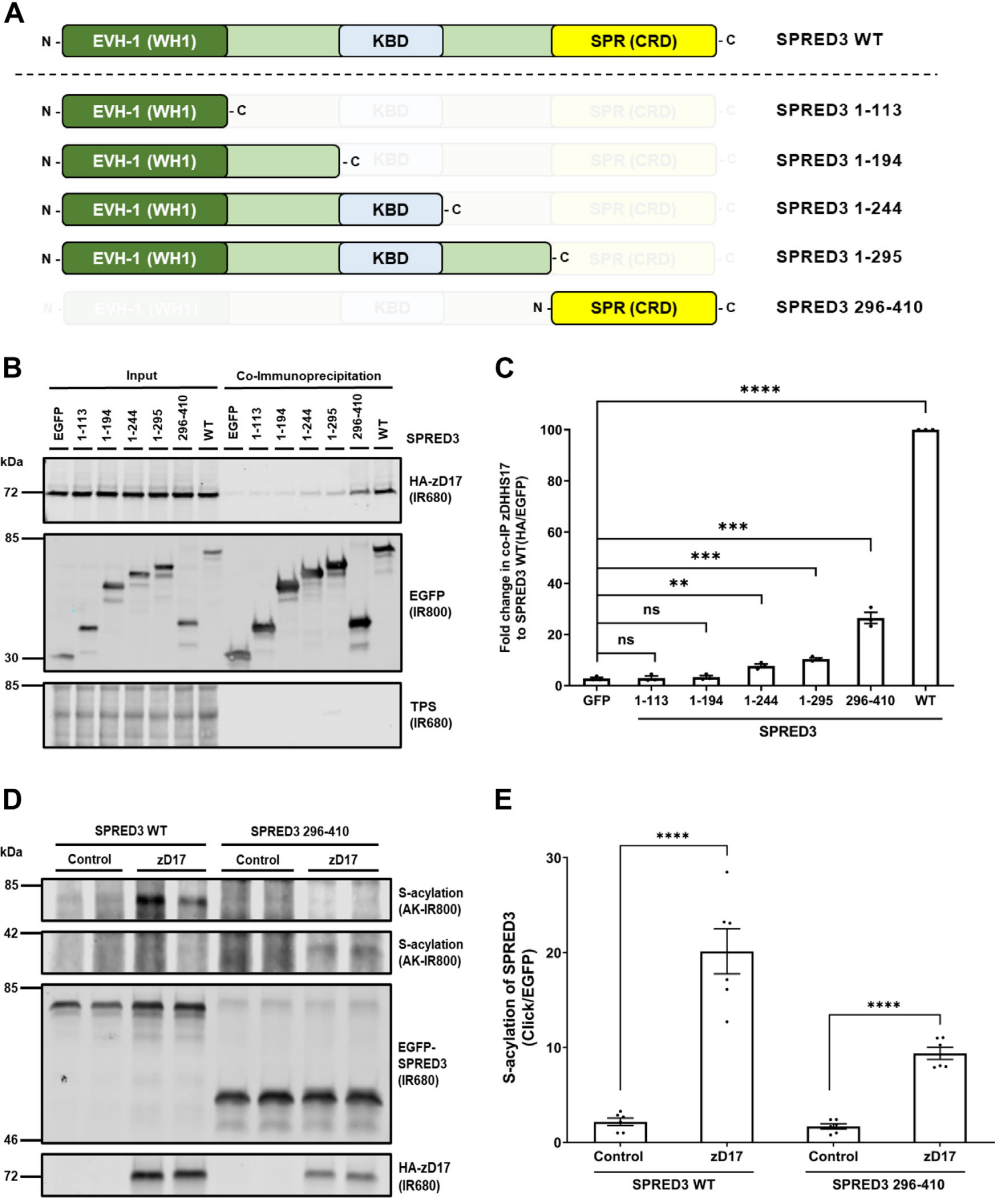
**The SPR domain of SPRED3 is sufficient for binding to, and S-acylation by, zDHHC17.***A*, schematic representation of SPRED3 truncation mutant constructs used in click chemistry and coimmunoprecipitation assays: SPRED3 WT, 1 to 113, 1 to 194, 1 to 244, 1 to 295, and 296 to 410 (UniProt KB - Q2MJR0). EVH-1, Ena/VASP (Enabled/vasodilator-stimulated phosphoprotein) Homology 1 domain, also known as WH1 for WASP (Wiskott–Alsrich Syndrome Protein) homology 1 domain; KBD, c-Kit kinase binding domain; SPR, Sprouty domain, which is also referred to as CRD (Cysteine-rich domain). All constructs used have EGFP tags appended at the N terminus. *B*, HEK293T cells were cotransfected with HA-tagged zDHHS17 (a catalytically inert form of the enzyme) along with plasmids encoding for EGFP-tagged SPRED3 1 to 113, 1 to 194, 1 to 244, 1 to 295 or 296 to 410, or EGFP alone (as a control). Cell lysates were incubated with agarose beads conjugated to an EGFP antibody and coimmunoprecipitated proteins were analyzed by immunoblotting. Representative images showing zDHHS17 (*top; IR680*) and SPRED3 (*middle; IR800*) detected in input and immunoprecipitated samples on the same immunoblot. A total protein stain (TPS) is also shown (*bottom panel; IR680*). The positions of the molecular weight markers are shown on the *left* side of all immunoblots. *D*, HEK293T cells were transfected with plasmids encoding EGFP-tagged SPRED3 WT or SPRED3 296 to 410, together with either pEF-BOS-HA (referred to as “control” in the figure) or HA-zDHHC17. Cells were incubated with 100 μM palmitic acid azide (C16:0-azide) for 4 h and labeled proteins reacted with alkyne (AK) IRdye-800 nm. Representative images showing SPRED3 S-acylation (*top; AK-IR800*) and SPRED3 levels (*middle; IR680*) detected on the same immunoblot. For zDHHC17, HA (*bottom; IR680*) was revealed for the same samples on a different immunoblot. The positions of the molecular weight markers are shown on the *left* side of all immunoblots. *C*, graph showing the mean fold change in coimmunoprecipitated zDHHS17 after normalization against the SPRED3 WT. Error bars represent ± SEM; each replicate is shown with *filled circles*. Differences were analyzed by unpaired *t* test compared to the EGFP control. ∗∗∗∗ denotes *p* < 0.0001, ∗∗∗*p* < 0.001, ∗∗*p* < 0.01, ns = non significant (n = 3 for three independent experiments). *E*, graph showing mean SPRED3 S-acylation levels after normalization against the corresponding control samples. Error bars represent ± SEM; each replicate is shown with *filled circles*. Differences were analyzed by unpaired *t* test to the control for each substrate (∗∗∗∗ denotes *p* < 0.0001, ∗∗∗*p* < 0.001, ∗∗*p* < 0.01, n = 6 from three independent experiments).

To further understand how zDHHC17 can recognize and S-acylate substrate proteins without zDABM interactions, we further analyzed SPRED3 as we predict that this protein only has a single zDHHC17-binding site (Fig. 5, *F* and *G*). To explore the features required for SPRED3 interaction with zDHHC17, we designed a series of SPRED3 truncation mutants in which specific domains were removed (see Fig. 6*A*). Interestingly the isolated SPR domain (amino acids 296–410) was able to robustly coprecipitate zDHHC17 (catalytically dead) albeit at reduced levels compared to WT SPRED3 (Fig. 6, *B* and *C*), consistent with this region of SPRED3 containing a zDHHC17-binding site. We did also detect slight but significant binding of 1 to 244 and 1 to 295 SPRED3 truncation mutants to zDHHC17 in this assay, perhaps suggesting an additional weaker binding site upstream of the SPR domain (Fig. 6, *B* and *C*). Nevertheless, the SPR domain alone was efficiently S-acylated by zDHHC17 in click chemistry assays (Fig. 6, *D* and *E*), thus showing that interaction of the enzyme with this region of SPRED3 is sufficient for S-acylation.

### SPRED3 binding to zDHHC17 does not require the ANK domain or the C terminus

**Figure 7:**
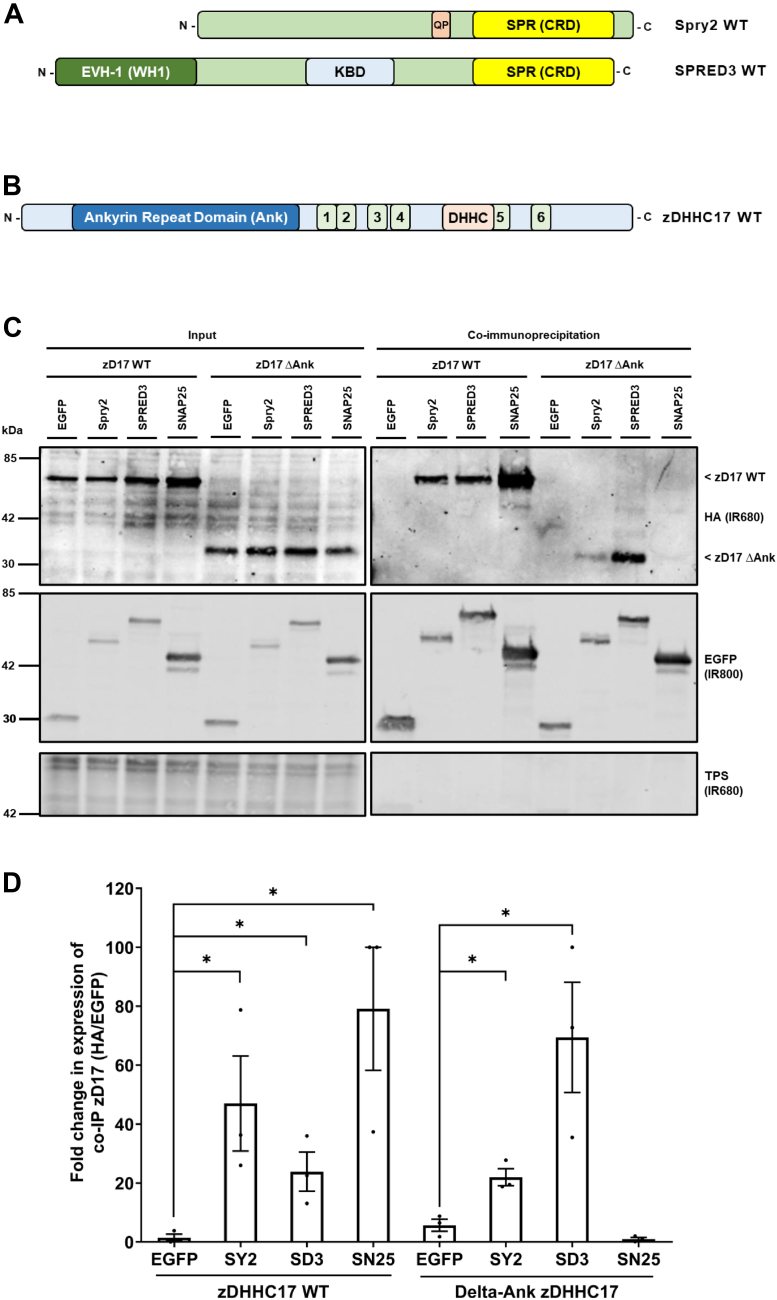
**Spry2 and SPRED3 can both effectively bind zDHHC17 in the absence of the ankyrin repeat domain.***A*, schematic diagram comparing Spry2 and SPRED3 constructs. EVH-1, Ena/VASP (Enabled/vasodilator-stimulated phosphoprotein) Homology 1 domain also known as WH1, WASP (Wiskott-Alsrich Syndrome Protein) homology one domain; KBD, c-Kit kinase binding domain; QP indicates the zDABM position; SPR, Sprouty domain also referred to as CRD (Cysteine-rich domain). All constructs used have EGFP tags appended at the N terminus. *B*, schematic diagram of zDHHC17 WT. Ankyrin repeat domain; Ank; transmembrane domains; 1–6; DHHC domain; DHHC. All constructs have HA tags appended at the N terminus. zDHHC17 WT (aa 11–632); zDHHC17 ΔAnk (aa 287–632); zDHHC17 ΔC (aa 11–569). *C*, HEK293T cells were cotransfected with HA-tagged zDHHC17 WT or zDHHC17 ΔAnk (Ankyrin repeat domain removed) along with plasmids encoding EGFP-tagged Spry2, SPRED3, SNAP25, or EGFP alone (as a control). Cell lysates were incubated with agarose beads conjugated to an EGFP antibody and coimmunoprecipitated proteins were analyzed by immunoblotting. Representative images showing zDHHC17 (*top; IR680*) and EGFP-tagged proteins (*middle; IR800*) in the input and immunoprecipitated samples detected on the same immunoblot. A total protein stain (TPS) is also shown (*bottom panel; IR680*). The positions of the molecular weight markers are shown on the *left* side of all immunoblots. *D*, graph showing the fold change in coimmunoprecipitated zDHHC17 after normalization. SY2 = Spry2, SD3 = SPRED3, SN25 = SNAP25. Error bars represent ± SEM; each replicate is shown with *filled circles*. Differences were analyzed by unpaired *t* test compared to the EGFP control (∗ denotes *p* < 0.05, n = 3, from three independent experiments).

**Figure 8:**
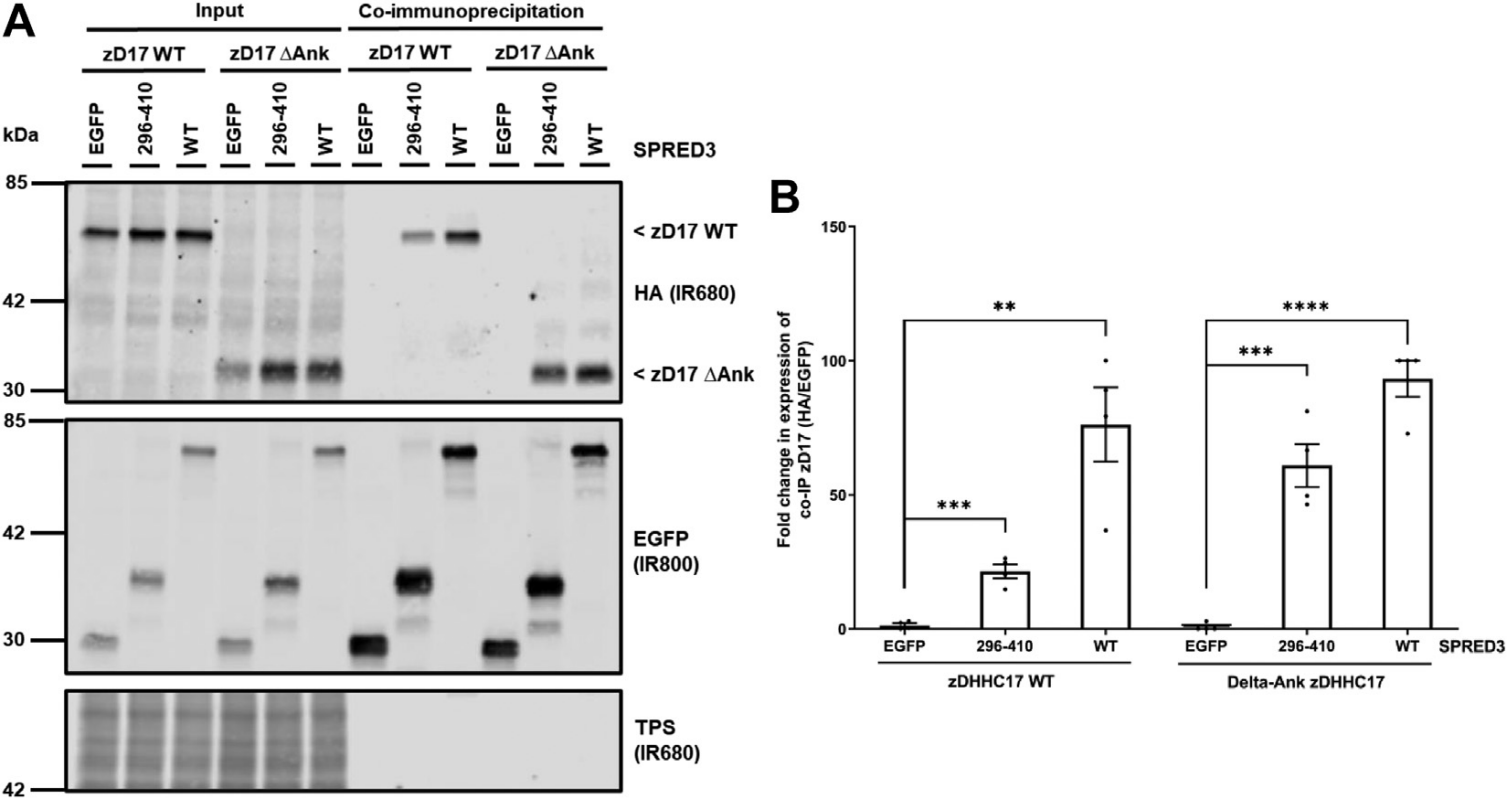
**The SPR domain of SPRED3 can bind a zDHHC17 mutant that lacks the ankyrin repeat domain.***A*, HEK293T cells were cotransfected with HA-tagged zDHHC17 WT or zDHHC17 ΔAnk (Ankyrin repeat domain removed) along with plasmids encoding EGFP-tagged SPRED3 WT, SPRED3 296 to 410 or EGFP alone (as a control). Cell lysates were incubated with agarose beads conjugated to an EGFP antibody and coimmunoprecipitated proteins were analyzed by immunoblotting. Representative images showing coimmunoprecipitated zDHHC17 (*top; IR680*) and SPRED3 levels (*middle; IR800*) detected on the same immunoblot. A total protein stain (TPS) is also shown (*bottom panel; IR680*). The positions of the molecular weight markers are shown on the *left* side of all immunoblots. *B*, graph showing the mean fold change in coimmunoprecipitated zDHHC17 after normalization. Error bars represent ± SEM; each replicate is shown with *filled circles*. Differences were analyzed by unpaired *t* test compared to the EGFP control (∗∗∗∗ denotes *p* < 0.0001, ∗∗∗*p* < 0.001, ∗∗*p* < 0.01, n = 4 from two independent experiments).

As SPRED3 interaction with zDHHC17 involves the SPR domain of the substrate protein rather than a zDABM, we next investigated if SPRED3 interacts with ANK17 as reported for other substrates of zDHHC17. For this analysis, HA-tagged zDHHC17 or a mutant lacking the ANK domain were coexpressed with either EGFP-SPRED3, EGFP-Spry2, or EGFP-SNAP25; the latter protein has previously been shown to require an intact zDABM for S-acylation by zDHHC17 (23). The EGFP-tagged proteins were then captured on GFP trap beads and the coimmunoprecipitation of WT or ΔANK mutant HA-zDHHC17 quantified. Interestingly, we found that whereas SNAP25 failed to coprecipitate ΔANK zDHHC17, Spry2 still interacted with this construct albeit with reduced efficiency, and SPRED3 displayed similar binding to the WT and ΔANK zDHHC17 proteins (Fig. 7). This analysis suggests that the interaction of SPRED3 with zDHHC17 occurs outside of the ANK domain in the enzyme. The reduced but detectable binding of Spry2 to the ΔANK mutant is consistent with Spry2 having two binding sites, the major one being the zDABM interaction with the ANK domain and the second binding mode presumably being similar to the ANK domain–independent mode that occurs with SPRED3. We also confirmed that binding of the SPR domain of SPRED3 to zDHHC17, similar to the full-length SPRED3 proteins, was independent of the ANK domain (Fig. 8).

**Figure 9:**
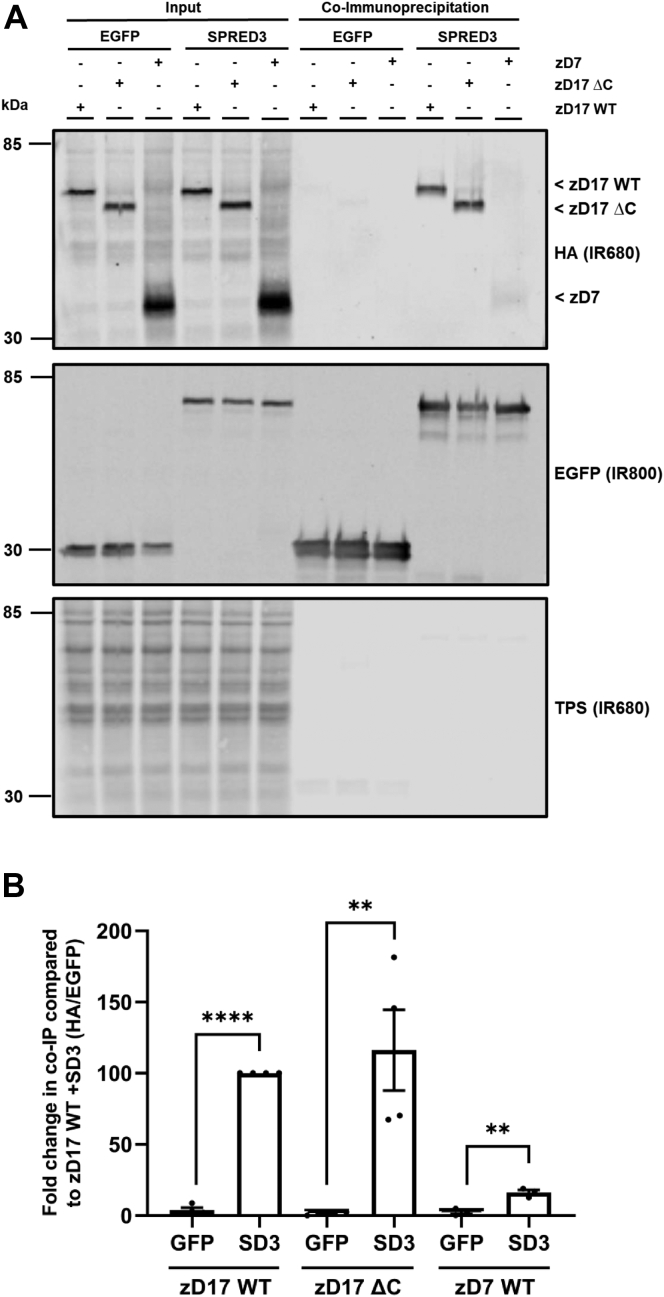
**SPRED3 can bind to a zDHHC17 mutant that lacks the C terminus and displays stronger binding to zDHHC17 than zDHHC7.***A*, HEK293T cells cotransfected with HA-tagged zDHHC17 WT, zDHHC17 ΔC (C-terminal end removed – see Fig. 7*B*; zDHHC17 ΔC; aa 11–569), or zDHHC7 WT along with plasmids encoding EGFP-tagged SPRED3 WT or EGFP alone (as a control). Cell lysates were incubated with agarose beads conjugated to an EGFP antibody and coimmunoprecipitated proteins were resolved by immunoblot. Representative images showing coimmunoprecipitated zDHHC17 or zDHHC7 (*top; IR680*), and EGFP-tagged protein levels (*middle; IR800*) detected on the same immunoblot. A total protein stain (TPS) is also shown (*bottom panel; IR680*). The positions of the molecular weight markers are shown on the *left* side of all immunoblots. *B*, graph showing the fold change in coimmunoprecipitated zDHHC17 WT, zDHHC17 ΔC, or zDHHC7 WT after normalization. SD3 = SPRED3. Error bars represent ± SEM; each replicate is shown with *filled circles*. Differences were analyzed by unpaired *t* test to each EGFP control (∗∗∗∗ denotes *p* < 0.0001 and ∗∗*p* < 0.01, n = 4 from three independent experiments).

Finally, we investigated if the binding of SPRED3 to zDHHC17 involved the C-terminal domain of the enzyme (*i.e.*, the region downstream of TM6; see Fig. 7*B*). In this experiment, we also assessed the binding of SPRED3 to a different enzyme, zDHHC7. The results of the coimmunoprecipitation assays in Figure 9 show that (i) removal of the C terminus of zDHHC17 does not affect interaction with SPRED3 and (ii) binding of SPRED3 to zDHHC7 was very weak and far lower than seen with zDHHC17, showing that although SPRED3 does not require the ANK and C-terminal domains of zDHHC17 for binding, the interaction is nevertheless specific for this enzyme isoform.

## Discussion

The results of this study uncover a striking difference in the S-acylation patterns of different proteins by zDHHC17. In contrast to analysis of SNAP25-zDHHC17 interactions (14, 15, 16, 21), the findings reported here for Spry2, SPRED1, SPRED2, and SPRED3 show that a simple zDABM interaction followed by substrate S-acylation model does not fit for all proteins. In fact, SNAP25, Spry2, and SPRED proteins each show a different profile, either (i) zDABM interaction with zDHHC17 is coupled to and essential for S-acylation (SNAP25); (ii) zDABM interaction with zDHHC17 occurs but the protein is not robustly S-acylated (SPRED1/2); (iii) zDABM interaction with zDHHC17 occurs but is dispensable for S-acylation (Spry2); and (iv) the substrate is S-acylated by zDHHC17 but lacks a zDABM sequence (SPRED3). How can these findings be interpreted? We previously reported that S-acylation of SNAP25 by zDHHC17 critically depends on the length of the linker region between the zDABM sequence and the S-acylated cysteines (13). Thus, we propose for Spry2 and SPRED1/2 that zDHHC17 interaction with the zDABM does not align the cysteine-rich region and the catalytic DHHC domain of zDHHC17 in an optimal position to allow S-acylation. Indeed, for SPRED3, we find that zDHHC17 interaction with the cysteine-rich SPR domain is coupled to S-acylation. We propose that a similar mechanism also operates for Spry2, as a truncation mutant containing the SPR domain (155–315) interacts with and is S-acylated by zDHHC17, despite lacking a zDABM. Interestingly, SPRED1 and SPRED2 did not show a significant increase in S-acylation by zDHHC17 in our experiments, suggesting either that zDHHC17 does not bind to the SPR domain of these proteins or alternatively that it does bind but subsequent S-acylation is inefficient. It is worth noting that previous work by Butland *et al.* (17) found that both SPRED1 and SPRED3 were significantly S-acylated by zDHHC17; however, there was a marked difference in S-acylation efficiencies: SPRED1 S-acylation was only increased by 1.35-fold, whereas SPRED3 S-acylation was increased by 8-fold. Therefore, the results of our study and the study of Butland *et al.* (17) are in broad agreement, albeit that S-acylation of SPRED1 by zDHHC17 did not reach statistical significance in our experiments. It will be interesting to explore the reasons of why SPRED3 is a better substrate of zDHHC17 than SPRED1/2. Perhaps different cysteine configurations of the SPR domains favor SPRED3 S-acylation over SPRED1/2 S-acylation.

Surprisingly, we also found that the ANK domain of zDHHC17 is dispensable for binding to SPRED3 and that Spry2 retains residual binding when the ANK domain is removed. Furthermore, we showed that the isolated SPR domain of SPRED3 also interacts with zDHHC17 lacking the ANK domain. This, to our knowledge, is the first demonstration of ANK domain–independent interaction of zDHHC17 linked to substrate S-acylation. In addition, the C terminus of zDHHC17 was also dispensable for SPRED3 binding. Based on these results, we propose that zDHHC17 interacts with SPRED3 through the cytosolic loop between TMDs 4 and 5, which contains the DHHC-CRD. If SPRED3 does bind to this region of zDHHC17, then it begs the question of whether this is a specific interaction as this region is common to all zDHHC enzymes. However, although we detected marginal binding of zDHHC7 to SPRED3 (Fig. 9), the interaction with zDHHC17 was substantially greater, implying that there is specificity in the zDHHC17-SPRED3 interaction. Work is currently ongoing in our group to more finely map the novel binding regions in both SPRED3 and zDHHC17.

Finally, it is worthwhile speculating on the relevance of the zDABM in Spry2 (and SPRED1/2). If this major binding mode is not linked to or required for S-acylation, what is its purpose? One possibility is that the zDABM-ANK17 interaction provides a means to regulate the timing of Spry2 S-acylation. For example, growth factor–dependent changes in Spry2 phosphorylation (or some other post-translational modification) might facilitate reorganization of Spry2–zDHHC17 complexes, moving from a zDABM-dependent interaction to an alternative binding mode that facilitates Spry2 S-acylation, enhanced stability, and plasma membrane targeting (19). Conversely, the different binding modes of Spry2 might exert a regulatory effect on zDHHC17 and in so-doing contribute to the regulation of cellular S-acylation dynamics. We are exploring these possibilities through knockdown and rescue experiments.

In conclusion, we have identified a novel mode of zDHHC17-substrate binding that is linked to S-acylation and further shown that the presence of zDABM sequence(s) in zDHHC17 substrates does not imply their role in S-acylation.

## Experimental procedures

### Plasmids

Mouse HA-tagged zDHHC17 and zDHHC7 were a kind gift of Dr Masaki Fukata (NIPS) (3). EGFP-tagged Spry2 and SPRED2 constructs are as previously described (19, 16). The ΔANK and ΔC mutants of zDHHC17 were previously described (12) N-terminally EGFP-tagged SPRED1 and SPRED3 (and associated mutants) were generated by Genscript UK.

### Antibodies

Mouse anti-EGFP (JL8) was purchased from Takara and used at a dilution of 1:4000 for immunoblotting. Rat high affinity anti-HA was provided by Sigma and used at a dilution of 1:1000 for immunoblotting.

### Cell culture and transfection

Human embryonic kidney 293T cells (HEK293T; ATCC #CRL2316) were cultured in Dulbecco's modified Eagle's medium (DMEM) media (Gibco) supplemented with 10% fetal bovine serum (Gibco). Rat adrenal phaeochromocytoma PC12 cells (PC12; ATCC #CRL-1721) were maintained in advanced RPMI-1640 media (Gibco) supplemented with 10% horse serum, 5% fetal bovine serum, and 1% L-glutamine (all from Gibco). Cells were plated on poly-D-lysine–coated 24-well plates or coverslips and incubated at 37 °C/5% CO_2_.

HEK293T and PC12 cells were transfected using PEI (24) and Lipofectamine 2000 reagent (Invitrogen Ltd), respectively, using a ratio of 2 μl PEI/Lipofectamine per μg DNA. For click chemistry assays, HEK293T cells were transfected with 0.33 μg of pEGFP-Spry2 and 0.66 μg of HA-zDHHC (or pEF-BOS-HA as a negative control) constructs per well of a 24-well plate. For immunoprecipitation assays, HEK293T cells were cotransfected with 0.4 μg of pEGFP-substrate constructs and 0.6 μg of HA-zDHHC constructs per well of a 24-well plate. HEK293T cells were used approximately 20 h post-transfection.

For confocal imaging experiments, PC12 cells were transfected with 0.2 μg of each plasmid per well of a 24-well plate that contained a poly-D-lysine–coated coverslip. PC12 cells were used approximately 44 h post-transfection.

### Click chemistry

Transfected HEK293T cells were washed in 500 μl of PBS per well and then incubated with 500 μl/well of serum-free DMEM containing 1 mg/ml of fatty acid–free bovine serum albumin and 100 μM of palmitic acid (C16:0) azide (25) for 4 h at 37 °C and 5 % CO_2_. After washing with PBS, cells were lysed on ice using 100 μl/well of lysis buffer (50 mM Tris pH 8; 0.5% SDS; 1× protease inhibitor cocktail) and transferred into fresh tubes. For each 100 μl of cell lysate, 80 μl of fresh click reaction mix containing 2.5 μM of alkyne dye-IR800, 2 mM of CuSO_4_ (Copper (II) Sulfate), 0.2 mM of TBTA (Tris[(1-benzyl-1H-1,2,3-triazole-4yl) methyl]), and 20 μl of 4 mM ascorbic acid was added. The samples were then vortexed and incubated for 1 h with end-over-end rotation at room temperature (RT). Sixty-seven microliters of 4× Laemmli sample buffer (100 mM DTT) was added to each sample and heated at 95 °C for 5 min. Samples were resolved by SDS-PAGE and HA and EGFP-tagged proteins detected by immunoblotting. Click chemistry in PC12 cells was performed on GFP-trap beads following immunoprecipitation of Spry2 (see following section).

### Immunoprecipitation assays

Transfected HEK293T cells were scraped from the surface of the 24-well plate in 200 μl of lysis buffer (PBS, 0.5% Triton X-100, 1× protease inhibitors) and incubated on ice for 30 min. The lysate was clarified at 14,000*g* for 5 min at 4 °C and supernatant from this step was collected in fresh tubes, supplemented with 300 μl of PBS to a final volume of 500 μl. Fifty microliter aliquots were kept as *Input* samples and the remaining 450 μl of cell lysate was mixed with 10 μl bed volume of GFP-Trap beads (Chromotek) and incubated for no less than 1 h with end-over-end rotation at 4 °C. Beads were recovered by centrifugation and washed in PBS. Fifty microliters of 2× Laemmli sample buffer (50 mM DTT) was then added, and bound proteins were eluted by heating the beads at 95 °C for 10 min. Supernatants were collected in fresh tubes as *Bound* samples and resolved by SDS-PAGE, followed by detection of HA and EGFP-tagged proteins by immunoblotting. For experiments in PC12 cells, the click reaction was performed on immunoprecipitates on GFP trap beads before elution in Laemmli sample buffer.

### Confocal microscopy

Transfected PC12 cells plated on poly-D-lysine–coated coverslips were washed in PBS and cells were fixed in 4% formaldehyde (Pierce, Thermo Fisher Scientific) and incubated for 30 min at RT. The coverslips were then washed in PBS, air-dried, and subsequently mounted on glass slides using Mowiol mounting agent. All images were acquired as z-stacks using an SP8 confocal microscope (Leica Microsystems).

### Data quantification and statistical analysis

Quantification of band intensity obtained from immunoblots/gels was performed using Odyssey Infrared Imaging System Data Quantification (LI-COR Inc).

Statistical analyses were performed in GraphPad Prism 9.0 (GraphPad Software Inc). Differences were analyzed by unpaired *t* test as specified in figure legends.

All graphs were generated with GraphPad Prism 9.0. Mean values with SEM are plotted, and the number of replicates is indicated in the figure legends. For significant results, ∗∗∗∗ denotes *p* < 0.0001, ∗∗∗*p* < 0.001, ∗∗*p* < 0.01, ∗*p* < 0.05, and ns *p* > 0.05.

## Data availability

All data is contained within the article.

## Conflict of interest

The authors declare that there are no conflicts of interest associated with this paper.

## References

[1] Chamberlain L.H., Shipston M.J. (2015). The physiology of protein S-acylation. Physiol. Rev..

[2] Main A., Fuller W. (2021). Protein S-palmitoylation: advances and challenges in studying a therapeutically important lipid modification. FEBS J..

[3] Fukata M., Fukata Y., Adesnik H., Nicoll R.A., Bredt D.S. (2004). Identification of PSD-95 Palmitoylating enzymes. Neuron.

[4] Lobo S., Greentree W.K., Linder M.E., Deschenes R.J. (2002). Identification of a Ras palmitoyltransferase in Saccharomyces cerevisiae. J. Biol. Chem..

[5] Roth A.F., Feng Y., Chen L., Davis N.G. (2002). The yeast DHHC cysteine-rich domain protein Akr1p is a palmitoyl transferase. J. Cell Biol..

[6] Roth A.F., Wan J., Bailey A.O., Sun B., Kuchar J.A., Green W.N. (2006). Global analysis of protein palmitoylation in yeast. Cell.

[7] Rana M.S., Kumar P., Lee C.J., Verardi R., Rajashankar K.R., Banerjee A. (2018). Fatty acyl recognition and transfer by an integral membrane S-acyltransferase. Science.

[8] Rana M.S., Lee C.J., Banerjee A. (2019). The molecular mechanism of DHHC protein acyltransferases. Biochem. Soc. Trans..

[9] Malgapo M.I.P., Linder M.E. (2021). Substrate recruitment by zDHHC protein acyltransferases. Open Biol..

[10] Rodenburg R.N.P., Snijder J., van de Waterbeemd M., Schouten A., Granneman J., Heck A.J.R. (2017). Stochastic palmitoylation of accessible cysteines in membrane proteins revealed by native mass spectrometry. Nat. Commun..

[11] Singaraja R.R., Hadano S., Metzler M., Givan S., Wellington C.L., Warby S. (2002). HIP14, a novel ankyrin domain-containing protein, links huntingtin to intracellular trafficking and endocytosis. Hum. Mol. Genet..

[12] Lemonidis K., Gorleku O.A., Sanchez-Perez M.C., Grefen C., Chamberlain L.H. (2014). The Golgi S-acylation machinery is comprised of zDHHC enzymes with major differences in substrate affinity and S-acylation activity. Mol. Biol. Cell.

[13] Salaun C., Greaves J., Tomkinson N.C.O., Chamberlain L.H. (2020). The linker domain of the SNARE protein SNAP25 acts as a flexible molecular spacer that ensures efficient S-acylation. J. Biol. Chem..

[14] Lemonidis K., Sanchez-Perez M.C., Chamberlain L.H. (2015). Identification of a novel sequence motif recognised by the ankyrin-repeat domain of zDHHC17/13 S-acyl-transferases. J. Biol. Chem..

[15] Verardi R., Kim J.S., Ghirlando R., Banerjee A. (2017). Structural basis for substrate recognition by the ankyrin repeat domain of human DHHC17 palmitoyltransferase. Structure.

[16] Lemonidis K., MacLeod R., Baillie G.S., Chamberlain L.H. (2017). Peptide array based screening reveals a large number of proteins interacting with the ankyrin repeat domain of the zDHHC17 S-acyltransferase. J. Biol. Chem..

[17] Butland S.L., Sanders S.S., Schmidt M.E., Riechers S.P., Lin D.T., Martin D.D. (2014). The palmitoyl acyltransferase HIP14 shares a high proportion of interactors with huntingtin: implications for a role in the pathogenesis of Huntington's disease. Hum Mol Genet..

[18] Huttlin E.L., Bruckner R.J., Paulo J.A., Cannon J.R., Ting L., Baltier K. (2017). Architecture of the human interactome defines protein communities and disease networks. Nature.

[19] Locatelli C., Lemonidis K., Salaun C., Tomkinson N.C.O., Chamberlain L.H. (2020). Identification of key features required for efficient S-acylation and plasma membrane targeting of sprouty-2. J. Cell Sci..

[20] Masoumi-Moghaddam S., Amini A., Morris D.L. (2014). The developing story of Sprouty and cancer. Cancer Metastasis Rev..

[21] Greaves J., Prescott G.R., Fukata Y., Fukata M., Salaun C., Chamberlain L.H. (2009). The hydrophobic cysteine-rich domain of SNAP25 couples with downstream residues to mediate membrane interactions and recognition by DHHC palmitoyl transferases. Mol. Biol. Cell.

[22] Greaves J., Salaun C., Fukata Y., Fukata M., Chamberlain L.H. (2008). Palmitoylation and membrane interactions of the neuroprotective chaperone cysteine-string protein. J. Biol. Chem..

[23] Greaves J., Gorleku O.A., Salaun C., Chamberlain L.H. (2010). Palmitoylation of the SNAP25 protein family: specificty and regulation by DHHC palmitoyl transferases. J. Biol. Chem..

[24] Longo P.A., Kavran J.M., Kim M.S., Leahy D. (2013). Transient mammalian cell transfection with polyethylenimine (PEI). Methods Enzymol..

[25] Greaves J., Munro K.R., Davidson S.C., Riviere M., Wojno J., Smith T.K. (2017). Molecular basis of fatty acid selectivity in the zDHHC family of S-acyltransferases revealed by click chemistry. Proc. Natl. Acad. Sci. U. S. A..

